# Human Papillomavirus (HPV) Vaccine Knowledge, Awareness and Acceptance among Dental Students and Post-Graduate Dental Residents

**DOI:** 10.3390/dj8020045

**Published:** 2020-05-09

**Authors:** Steven Kent Mann, Karl Kingsley

**Affiliations:** 1 Department of Advanced Education in Pediatric Dentistry, School of Dental Medicine, University of Nevada, Las Vegas, 1700 West Charleston Blvd, Las Vegas, NV 89106, USA; manns4@unlv.nevada.edu; 2 Department of Biomedical Sciences, School of Dental Medicine, University of Nevada, Las Vegas, 1001 Shadow Lane, Las Vegas, NV 89106, USA

**Keywords:** human papillomavirus (HPV), vaccination, dental student, post-graduate dental resident

## Abstract

Introduction: The recent development of a vaccine that is highly effective against the human papillomavirus (HPV) has been met with widespread clinical and public health professional acceptance. However, social and societal barriers to vaccination may hamper public health efforts to prevent HPV-mediated diseases. Although a few studies have evaluated knowledge or awareness of HPV vaccination among dentists or dental educators, few studies have evaluated the acceptance, knowledge and awareness of HPV vaccination among dental students and post-graduate dental residents. The primary goal of this study is to evaluate survey responses regarding acceptance, knowledge and awareness of HPV vaccination among dental students and post-graduate dental residents. Methods: This study was a retrospective analysis of a previously administered and collected questionnaire. The original protocol was reviewed by the UNLV Biomedical Institutional Research Board (IRB) and was deemed excluded from IRB review (OPRS#0811-2911). Results: Two hundred and ninety-three (*N* = 293) dental student and forty-one (*N* = 41) post-graduate dental resident questionnaires were available for a total sample size of *N* = 334. In brief, although the majority of dental students and residents agreed that vaccines are safe and effective, less than half of dental students (37.5%) or dental residents (48.7%) had discussed the HPV vaccine with a physician or had received the vaccine themselves. In addition, a significant percentage of dental students and residents felt they did not have enough information regarding the HPV vaccine (25.6% and 26.8%, respectively) or had significant concerns about the side effects (17.1%). Conclusions: The data suggest more specific information in dental school microbiology and immunology courses might be needed to increase awareness and knowledge of the safety and effectiveness of vaccines, including the HPV vaccine. This enhanced education might also serve as a curricular focal point to answer questions regarding vaccine-related side effects and provide a mechanism for answering important questions regarding this vaccine.

## 1. Introduction

The recent development of a highly effective vaccine against the human papillomavirus (HPV) has been met with widespread clinical and public health interest and awareness based on scientific and lay literature reports of its effectiveness and resulting reductions in associated cancer risk [1,2,3,4]. Systematic reviews of randomized controlled trials (RCT) revealed strong antibody responses from two- and three-dose vaccinations that have resulted in a significant reduction in the incidence of external genital lesions and will likely result in long-term reduction in the incidence of high-risk cervical cancers and other HPV-mediated pathologies [5,6,7]. Recent systematic reviews and meta-analyses have revealed compelling evidence of substantial reductions in HPV-specific infections in geographic areas with high vaccination coverage, which will likely facilitate both direct clinical protection and more far-reaching effects involving herd immunity within those communities [8,9].

However, in spite of these positive reports of HPV vaccine effectiveness, social and societal barriers to vaccination may hamper public health efforts to prevent HPV-mediated diseases [10]. For example, the challenge of increasing awareness, knowledge and compliance among boys and young men has been particularly difficult as described by low participation rates since HPV has been viewed as a pathogen primarily affecting women [11]. In addition, cultural beliefs and religious barriers based on pre-marital sexual relations bans may also contribute to vaccine hesitancy among some specific sub-populations that may harbor negative views regarding the topic of preventing sexually transmitted infections [12]. Finally, the debunked myths and misconceptions regarding a link between vaccination and childhood autism and other pediatric pathologies may still be pervasive among a significant proportion of the population [13,14].

Although a few studies have evaluated knowledge or awareness of HPV vaccination among practicing dentists or dental educators, it is unclear whether this information receives sufficient emphasis in dental education to mold student views on vaccination that lead to their implementation by students when they enter dental practice. Few studies have evaluated the awareness, knowledge and acceptance of HPV vaccination among dental students and post-graduate residents [15,16]. The few studies to date that have evaluated dental students specifically, have found deficiencies in both knowledge and awareness that may be addressed through changes to the dental curriculum [17,18]. In fact, recent evidence has suggested that HPV-specific immunization training may be sufficient to increase student knowledge and increase the likelihood of recommending HPV immunization among dental hygiene students [19].

Based upon this information, the primary goal of this study is to evaluate dental student and post-graduate resident awareness, knowledge and acceptance of HPV vaccination during a curricular module devoted to immunization practices and HPV-specific vaccination.

## 2. Methods

### 2.1. Study Approval

The original protocol was reviewed by the UNLV Biomedical Institutional Research Board (IRB) and was deemed excluded from IRB review (OPRS#0811-2911) November 12, 2008. Informed consent was waived pursuant to the exemption to human subjects research under the Basic HHS Policy for Protection of Human Research Subjects, (46.101) Subpart A (b) regarding IRB exemption for research involving the use of education tests (cognitive, diagnostic, aptitude, achievement) in which the subjects cannot be identified directly or through identifiers.

In brief, this study was a retrospective analysis of previously administered and collected student responses. All enrolled dental students (*n* = 312) and post-graduate residents (*n* = 48) were asked to complete a voluntary questionnaire, based upon a previously validated HPV vaccine awareness, knowledge, perceptions and clinical practice survey discussed within the context of the required Microbiology and Immunology course session focused on HPV and HPV vaccination [20].

Students were then placed into small groups of four and asked to pick one group of four questions (Questions 3–6 regarding general vaccine safety and efficacy or Questions 14–17 regarding HPV vaccine specifically) and find evidence-based information to support their answers. Each student would take one of the four selected questions and find peer-reviewed articles to support (or refute) their answers. The students then answered questions on a separate survey regarding how and where they found this peer-reviewed or evidence-based information (information literacy skills). Neither the HPV questionnaire or information literacy survey had any identifying information regarding the respondents. At the completion of this exercise, students were allowed to voluntarily turn in either the HPV questionnaire, the information literacy survey or both. The information from the literacy survey will be reviewed and published separately.

### 2.2. Questionnaire

The full questionnaire consisted of *n* = 17 total questions, divided between two sections. The first section consisted of eight non-specific vaccine related questions assessing general knowledge, awareness, perceptions and clinical practice guidelines. Four possible responses were available (Disagree, Neutral, Agree, Not applicable). These questions included:

1. Vaccines are necessary to protect public health
2. There are too many required vaccines
3. Vaccines are generally safe
4. Vaccination can make you sick
5. Some vaccines are dangerous
6. Vaccines are generally effective
7. I follow the vaccine guidelines for myself
8. I adhere to the vaccine guidelines for my family The second section was comprised of nine HPV-specific questions, which included:
9. I am aware of a vaccine for human papillomavirus (HPV)
10. HPV vaccination is important for me
11. HPV vaccination is important for (my) spouse/partner
12. HPV vaccination is important for (my) daughter(s)
13. HPV vaccination is important for (my) son(s)
14. I have discussed HPV vaccination with a doctor
15. I do not have enough information about the HPV vaccine
16. I am concerned about possible HPV vaccine side effects
17. I have already received the HPV vaccine

### 2.3. Statistical Analysis

Compliance with IRB and Informed Consent exemption required that no demographic or other personal information could be collected at the time of the questionnaire collection, therefore no summary statistics or analysis for these participants and the corresponding responses can be provided. All questionnaire responses were transcribed in an Excel spreadsheet and descriptive statistics for the percentage of responses from each question were reported. Differences in responses between dental (DMD-level) students and post-graduate residents were analyzed using Chi square analysis and on-line software from GraphPad (San Diego, CA, USA).

## 3. Results

The voluntary questionnaire was administered to all enrolled dental (DMD) students (*n* = 312). Almost three hundred students completed this questionnaire, yielding an overall response rate of 93.9% (*n* = 293/312). The overall demographic breakdown of the overall student population was approximately 55% male and 45% female (Table 1), which was not significantly different from the DMD survey respondents, *p* = 0.7504. The racial and ethnic background of these students was approximately half White/Caucasian (49.4%) and half non-White minorities (50.6%), including 39.1% Asians and 8.7% Hispanics. These percentages were also not significantly different from the DMD survey respondents, *p* = 0.5692.

The voluntary questionnaire was also administered to all post-graduate dental (Pediatric, Orthodontic) residents (*n* = 48). Forty-one residents completed this questionnaire, yielding an overall response rate of 85.4% (*n* = 41/48). The overall demographic breakdown of the overall post-graduate resident population was approximately 48% male and 52% female (Table 2), which was not significantly different from the survey respondents, *p* = 0.6128. The racial and ethnic background of these students is approximately half White/Caucasian (46.3%) and half non-White minorities (54.2%), exclusively Asian. These percentages were also not significantly different from the survey respondents, *p* = 0.7512.

**Figure 1:**
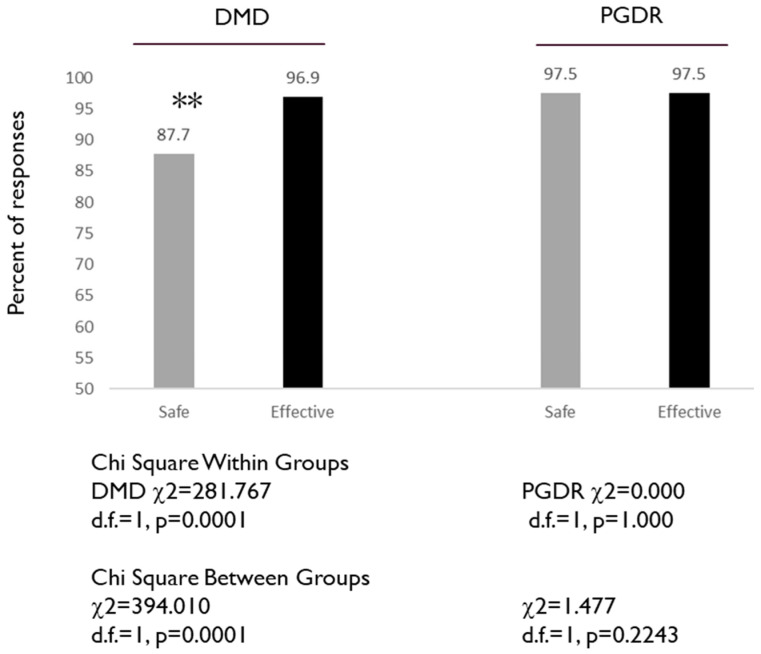
Assessment of DMD and PGDR responses to specific questions: Vaccines are generally safe, and Vaccination is generally effective. DMD students exhibited significantly lower percentages of agreeing that vaccines are safe (87.7%) versus effective (96.9%) *p* = 0.0001, while no differences were observed among the PGDR (97.5%), *p* = 1.000. Note: ** indicates statistically significant differences.

To assess the knowledge and awareness of positive aspects of vaccinations, including vaccine safety and efficacy, two specific questions from the questionnaire were evaluated: 3. Vaccines are generally safe, and 6. Vaccination is generally effective (Figure 1). Although the level of agreement was generally high, there was a significant difference in the percentage of DMD students that agreed vaccines are generally safe (87.7%) versus the percentage that agreed vaccines are generally effective (96.9%), *p* = 0.0001. The percentage of PGDR that agreed vaccines are generally safe (97.5%) was the same as the percentage that agreed vaccines are generally effective (97.5%), *p* = 1.000.

**Figure 2:**
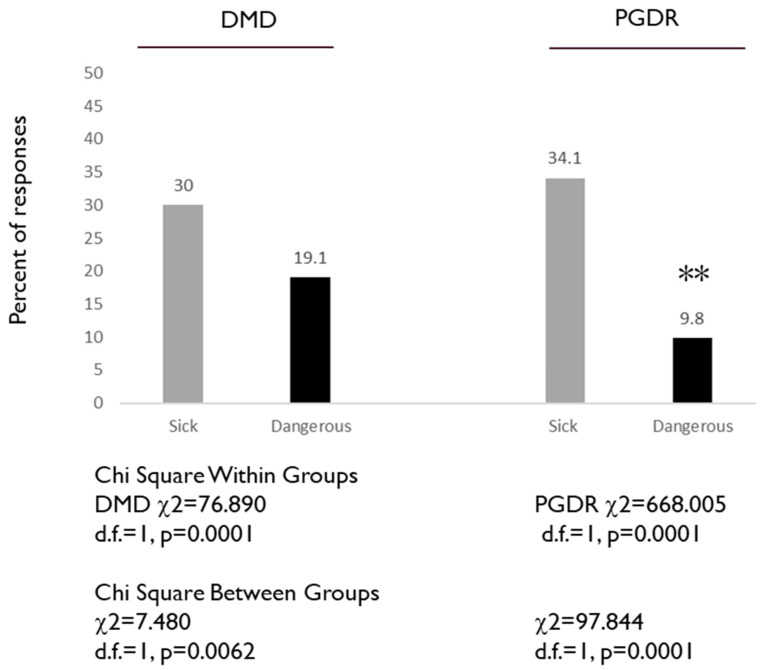
Assessment of DMD and PGDR responses to specific questions: Vaccines can make you sick, and some vaccines are dangerous. DMD students exhibited significantly higher percentages of agreeing that some vaccines are dangerous (19.1%) versus PGDR (9.8%) *p* = 0.0001, while responses to vaccines can make you sick were similar (30.0% versus 34.1%). Note: ** indicates statistically significant differences.

To assess the knowledge and awareness of negative aspects of vaccinations, including vaccine-related illness and side effects, two specific questions from the questionnaire were evaluated: 4. Vaccines can make you sick, and 5. Some vaccines are dangerous (Figure 2). There was a significant difference in the percentage of DMD students that agreed vaccines can make you sick (30.0%) and the percentage that agreed that some vaccines are dangerous (19.1%), *p* = 0.0001. Similarly, the percentage of PGDR that agreed vaccines can make you sick (34.1%) was higher and significantly different from the percentage who agreed that some vaccines are dangerous (9.8%), *p* = 0.0001. Although the percentage of DMD students and residents who agreed vaccines can make you sick was similar (30.0% versus 34.1%), a significantly higher proportion of DMD students responded that some vaccines were dangerous (19.1% versus 9.8%), *p* = 0.0001.

**Figure 3:**
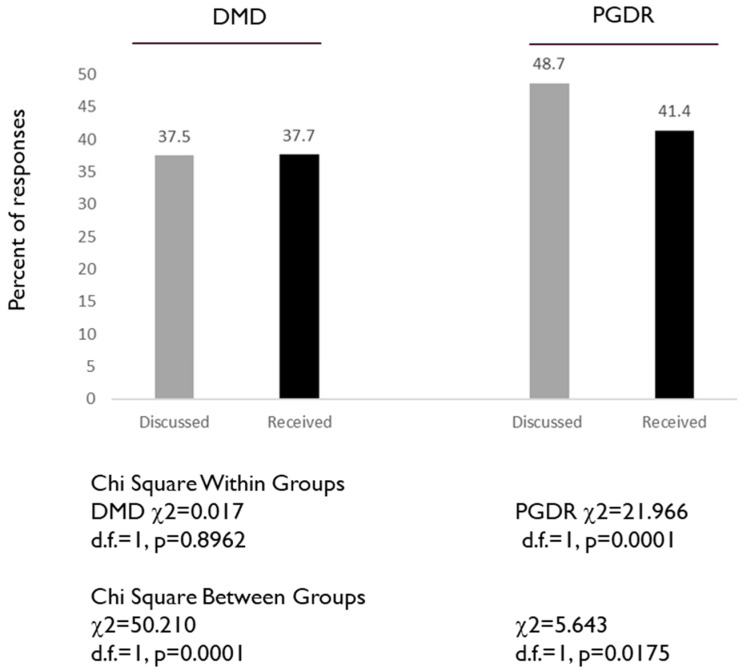
Assessment of DMD and PGDR responses to specific HPV-related questions: I have discussed HPV vaccination with a doctor, and I have received the HPV vaccination. DMD students exhibited similar percentages of having discussed HPV vaccination (37.5%) and having received HPV vaccination (37.7%), while PGDR exhibited higher percentages of having discussed HPV vaccination (48.7%) and having received the HPV vaccine (41.4%) *p* = 0.0001.

To assess specific aspects of HPV vaccination, two specific questions from the questionnaire were evaluated: 14. I have discussed HPV vaccination with a doctor, and 17. I have received the HPV vaccine (Figure 3). These data demonstrated that approximately one third (37.5%) of DMD students had discussed the HPV vaccination with a doctor, with a similar percentage reporting having received the HPV vaccination (37.7%), *p* = 0.8962. The percentage of PGDR that had discussed HPV vaccination with a doctor (48.7%) was significantly higher—and much higher than the percentage of DMD students—than the percentage that had received the HPV vaccination (41.4%), *p* = 0.0001.

**Figure 4:**
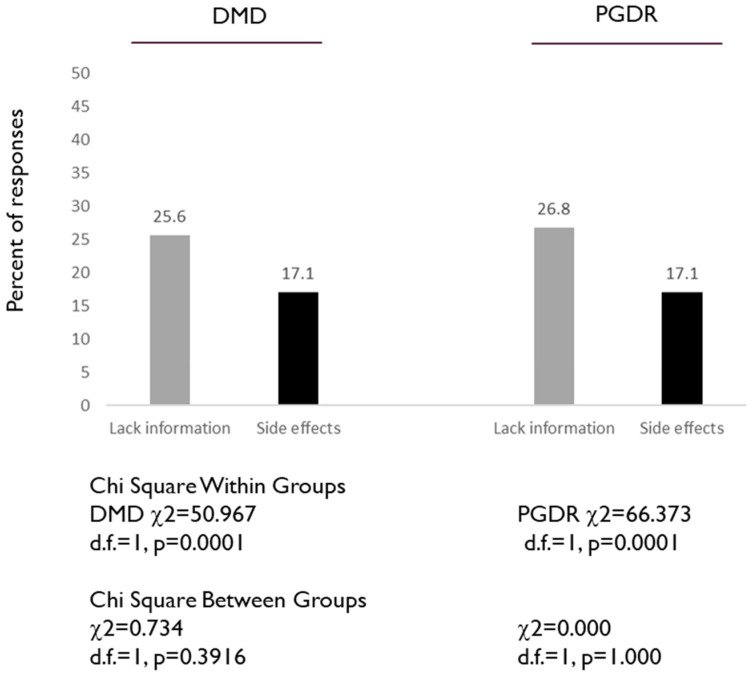
Assessment of DMD and PGDR responses to specific HPV-related questions: I do not have enough information about the HPV vaccine, and I am concerned about possible HPV vaccine side effects. Both groups (DMD, PGDR) had similar percentages of agreeing they did not have enough information regarding the HPV vaccine (25.6% and 26.8% respectively), *p* = 0.396. In addition, the same percentage of both the DMD and PGDR responders indicated they had concerns about possible HPV vaccine side effects (17.1%).

Finally, to assess the knowledge and awareness regarding HPV vaccinations, two additional questions from the questionnaire were evaluated: 15. I do not have enough information about this vaccine, and 16. I am concerned about possible HPV vaccine side effects (Figure 4). Analysis of these data revealed that approximately one quarter of both the DMD and PGDR agreed they did not have enough information regarding the HPV vaccine (25.6% and 26.8% respectively), which was not significantly different, *p* = 0.396. In addition, the same percentage of both the DMD and PGDR responders indicated they had concerns about possible HPV vaccine side effects (17.1%).

## 4. Discussion

The few previous studies that evaluated the awareness, knowledge and acceptance of HPV vaccination among dental students and post-graduate residents found deficiencies in both knowledge and awareness [15,16,17,18]. This study evaluated both dental student and post-graduate resident awareness, knowledge and acceptance of HPV vaccination during a curricular module devoted to immunization practices and HPV-specific vaccination and found noteworthy similarities and differences. For example, although the majority in both groups agreed vaccines were effective (~97%), significantly fewer dental students agreed they were safe. This provides support and confirmation of two previous studies that found that dental provider concerns about safety and efficacy corresponded with reluctance to discuss or recommend the HPV vaccine [21,22].

Moreover, the finding that nearly twice the percentage of dental students (approximately 20%) compared with dental residents (approximately 10%) agreed that some vaccines were dangerous may suggest that these concerns about vaccine safety, in general, may be more widespread and must be addressed within the dental school curriculum [23,24]. Although one recent study found that further education and training would improve willingness of dental providers to discuss and recommend HPV vaccination, previous work from the same group found dental student knowledge, awareness and willingness may be more malleable than established dental providers [17,18,25].

The findings that higher percentages of PGDR had discussed or received the HPV vaccination than DMD students may have several possible explanations. For example, the average age of a first year dental student is approximately 25 years old, while the average age for a post-graduate dental resident is closer to 30 years old—a difference of nearly five years that provides significant opportunities to interact with other healthcare providers and vaccination experts [26,27]. In addition, a higher proportion of PGDR may be married or partnered (likely also linked to age), which may increase the likelihood of interactions with healthcare providers recommending vaccinations, including the HPV vaccine [28,29].

This study also includes several limitations, which must also be considered. For example, this study is a retrospective analysis of previously collected data from an educational seminar and was therefore not designed specifically to collect demographic data and other variables that would allow for more in depth and comprehensive analysis of the respondents. In addition, more recent studies have developed and implemented psychometrically tested HPV surveys among college students, which may provide more accurate and useful information for comparison [30,31,32].

## 5. Conclusions

Despite these limitations, the data suggest more specific information in dental school microbiology and immunology courses might be needed to increase awareness and knowledge of the safety and effectiveness of vaccines, more specifically including the HPV vaccine and the relationship to oral cancer prevention. This enhanced education might also serve as a curricular focal point to answer questions regarding vaccine-related side effects and provide a mechanism for answering important questions regarding this vaccine.

## Figures and Tables

**Table 1:** DMD student population demographics.

Demographics	DMD Student Population	DMD Survey Respondents	Statistical Analysis
Sex			
Male	55.4% (*n* = 173/312)	54.9% (*n* = 161/293)	χ2 = 0.101, d.f. = 1
Female	44.6% (*n* = 139/312)	45.1% (*n* = 132/293)	*p* = 0.7504
Race/Ethnicity			
White	49.4% (*n* = 154)	48.5% (*n* = 242/293)	χ2 = 0.324, d.f. = 1
Non-White (Minority)	50.6% (*n* = 158)	51.5% (*n* = 151/293)	*p* = 0.5692
Asian	39.1% (*n* = 122)		
Hispanic	8.7% (*n* = 27)		
Other	2.9% (*n* = 9)		

**Table 2:** Post-graduate resident demographics.

Demographics	Post-Graduate Resident Population	Post-Graduate Survey Respondents	Statistical Analysis
Sex			
Male	47.9% (*n* = 23/48)	48.7% (*n* = 20/41)	χ2 = 0.256, d.f. = 1
Female	52.1% (*n* = 25/48)	51.3% (*n* = 21/41)	*p* = 0.6128
Race/Ethnicity			
White	45.8% (*n* = 22/48)	46.3% (*n* = 19/41)	χ2 = 0.101, d.f. = 1
Non-White (Minority)	54.2% (*n* = 26/48)	53.7% (*n* = 22/41)	*p* = 0.7512
Asian	54.2% (*n* = 26/48)	53.7% (*n* = 22/41)	
Hispanic	N/A	N/A	
Other	N/A	N/A	
